# From the Gastrointestinal Tract (GIT) to the Kidneys: Live Bacterial Cultures (Probiotics) Mediating Reductions of Uremic Toxin Levels via Free Radical Signaling

**DOI:** 10.3390/toxins5112042

**Published:** 2013-11-07

**Authors:** Luis Vitetta, Anthony W. Linnane, Glenda C. Gobe

**Affiliations:** 1 The University of Queensland, School of Medicine, Brisbane, St Lucia QLD 4072, Australia; 2 Medlab, Sydney, New South Wales, Australia; E-Mail: tlinnane.cmbm@live.com.au; 3 Monash University, Melbourne VIC 3800, Australia; 4 Centre for Kidney Disease Research, School of Medicine, Translational Research Institute at Princess Alexandra Hospital, The University of Queensland, Brisbane, St Lucia QLD 4072, Australia; E-Mail: g.gobe@uq.edu.au; 5 Department of Nephrology, Princess Alexandra Hospital, Ipswich Road, Woolloongabba, Brisbane QLD 4102, Australia

**Keywords:** toxins, commensal bacteria, uremia, chronic kidney disease, probiotics, reactive oxygen species (ROS)

## Abstract

A host of compounds are retained in the body of uremic patients, as a consequence of progressive renal failure. Hundreds of compounds have been reported to be retention solutes and many have been proven to have adverse biological activity, and recognized as uremic toxins. The major mechanistic overview considered to contribute to uremic toxin overload implicates glucotoxicity, lipotoxicity, hexosamine, increased polyol pathway activity and the accumulation of advanced glycation end-products (AGEs). Until recently, the gastrointestinal tract (GIT) and its associated micro-biometabolome was a neglected factor in chronic disease development. A systematic underestimation has been to undervalue the contribution of GIT dysbiosis (a gut barrier-associated abnormality) whereby low-level pro-inflammatory processes contribute to chronic kidney disease (CKD) development. Gut dysbiosis provides a plausible clue to the origin of systemic uremic toxin loads encountered in clinical practice and may explain the increasing occurrence of CKD. In this review, we further expand a hypothesis that posits that environmentally triggered and maintained microbiome perturbations drive GIT dysbiosis with resultant uremia. These subtle adaptation responses by the GIT microbiome can be significantly influenced by probiotics with specific metabolic properties, thereby reducing uremic toxins in the gut. The benefit translates to a useful clinical treatment approach for patients diagnosed with CKD. Furthermore, the role of reactive oxygen species (ROS) in different anatomical locales is highlighted as a positive process. Production of ROS in the GIT by the epithelial lining and the commensal microbe cohort is a regulated process, leading to the formation of hydrogen peroxide which acts as an essential second messenger required for normal cellular homeostasis and physiological function. Whilst this critical review has focused on end-stage CKD (type 5), our aim was to build a plausible hypothesis for the administration of probiotics with or without prebiotics for the early treatment of kidney disease. We postulate that targeting healthy ROS production in the gut with probiotics may be more beneficial than any systemic antioxidant therapy (that is proposed to nullify ROS) for the prevention of kidney disease progression. The study and understanding of health-promoting probiotic bacteria is in its infancy; it is complex and intellectually and experimentally challenging.

## 1. Introduction

The dysfunction of the kidneys leads to disturbed renal metabolism and to impaired glomerular filtration and tubular secretion/reabsorption problems. This results in the retention of toxic solutes, which affect all organs of the body. Chronic diseases such as cardiovascular disease and infections are key causes of morbidity and mortality among patients diagnosed with chronic kidney disease (CKD) [1]. It has been posited that toxins generated by gastrointestinal dysbiosis, and introduced into the body via the small and large bowel, may all contribute to CKD. They comprise advanced glycation end products, phenols and indoles [2]. Moreover, recent reports suggest that the bacterial load and the adverse products of the intestinal microbiota might influence chronic disease pathogenesis [3,4]. This is particularly relevant to the development of CKD, a disease of increasing prevalence in many Western societies. It has also been recently reported that the pharmacobiotic potential of the GIT micro-biometabolome may provide a plausible therapeutic avenue with the administration of live multi-strain probiotic cultures [5].

## 2. The GIT Microbial Metabolome

A recent analysis of gut microbial flora reported the occurrence of three dominant enterotypes characterized as *Bacteroides*, *Prevotella*, and *Ruminococcus* species [3,4]. Wu *et al.* [3] reported that the GIT microbiome is subject to modification by dietary and environmental variables as shown by altered patterns of enterotype dominance. They further reported that faecal communities clustered into the enterotypes *Bacteroides* and *Prevotella*. Enterotypes were strongly associated with long-term diets, particularly protein and animal fat (*Bacteroides*) *versus* carbohydrates (*Prevotella*). Furthermore, a controlled-feeding study of 10 subjects showed that microbiome composition changed detectably within 24 hours of initiating a high-fat/low-fiber or low-fat/high-fiber diet, but that enterotype identity remained stable during the 10-day study, which indicates that alternative enterotype states could be associated with long-term dietary patterns.

The mammalian gastrointestinal tract (GIT) microbiota extensively interact with the host via the intestinal mucosal surface, a site with a complex and interactional environment that is continuously exposed to a range of commensal microorganisms. Hence, the GIT is often subjected to intrinsic and extrinsic insults that can significantly disrupt GIT homeostasis. Recently, it was shown that renal toxicity from gut microbiota could be mediated by the biotransformation of melamine to cyanuric acid [6]. This finding serves to exemplify that the composition and metabolic activities of the gut microbiota oversee biometabolic interactions that can be beneficial (for example, vitamin production) or harmful (for example, toxins). 

## 3. GIT Dysbiosis and Uremic Toxins

As a consequence of the high metabolic load that is encountered in the GIT, toxins that arise due to adverse bacterial activity [7], or dietary and environmental influences [8], can exert a heavy toll on the enterocyte’s barrier function over a lifetime. Dysbiosis is a GIT perturbation that can follow the administration of antibiotics, imprudent dietary practices, immune deficits and pathogenic infections [9]. In conjunction with bacterial species that can broadly be categorized as saccharolytic (that is, those that predominantly ferment carbohydrates) or proteolytic (that is, those that predominantly utilize protein) [10], the enterocyte is exposed to an array of potentially toxic molecules that can predispose to uremia.

Precursors of uremic toxins are generated by GIT fermentation of amino acids like phenylalanine, tyrosine and tryptophan generating p-cresol, phenol and indole. These precursors are conjugated by passage through the intestinal wall and/or in the liver resulting in the circulating uremic retention solutes and/or toxins p-cresyl sulfate, p-cresyl glucuronide, phenyl sulfate, phenyl glucuronide, indoxyl sulfate and indoxyl glucuronide [8,9,10,11,12,13]. The biological impact of these molecules induces pro-inflammatory responses, leukocyte stimulation, and endothelial dysfunction [14,15,16,17]. The overproduction of pro-ory state in the GIT [11]. If a dysbiotic and inflamed GIT ensues [18], and is maintained [19], then triggers for CKD and end-stage renal disease (ESRD) become possible, thereby leading to the systemic spread of these molecules that then augments the likelihood of a uremic toxin overload for the kidneys [20]. A link is known between GIT pro-inflammatory responses, dysbiosis, and circulating uremic toxins; this connection is indicative of a GIT dysbiosis-renal relationship, particularly for the development of CKD.

The link between cardiovascular disease (CVD) and kidney disease suggests that uremic toxins are independent predictors of elevated CVD risk. Recent studies have demonstrated that even with a minor increase in serum p-cresyl sulfate (0.7 mg/L) an association with coronary artery disease was established [21]. Furthermore, experimental data have demonstrated that p-cresyl sulfate was causal for inflammation and damage to blood vessels [22]. Our group [2] have previously highlighted that the development of widespread obesity, metabolic syndrome (raised body mass index, blood pressure, blood glucose and triglyceride levels), insulin resistance and disrupted normal uric acid metabolism, most closely parallels the renal disease epidemic [23]. These combined factors from birth and over time may have a strong association with GIT dysbiosis supporting uremia over a lifetime.

## 4. Probiotics/Prebiotics and CKD

Robert Fuller (1989) [24] in early studies referred to probiotics as living organisms in food and dietary supplements that upon ingestion can improve the health of the host beyond their inherent basic nutritional make-up. Live probiotic cultures not only embrace those from different genera, such as *Bifidobacteria* and *Lactobacilli*, as well as different species of each genera, such as *Lactobacillus acidophilus, Lactobacillus bulgaricus* and *Lactobacillus rhamnosus*, but also different strains within a species, such as *Lactobacillus acidophilus* La-1 and *Lactobacillus acidophilus* North Carolina Fermentative Microorganism (NCFM). This fact emphasizes that different strains from the same species, while closely related metabolically, are not metabolically identical and may elaborate different physiological functions within the GIT [25], with different therapeutic outcomes on various disease states [26]. In contrast, prebiotics have been defined as those nutritional supplements (for example, inulin, a non-digestible plant oligosaccharide that is fermented by bacteria in the large bowel) that favours the growth and increasing survival of probiotic bacteria [26]. Live cultures of probiotic strains with established robust documented anti-inflammatory activity include *Lactobacillus paracasei* subsp. *paracasei*, *L. plantarum*, *L. acidophilus* and *Pediococcus pentosaceus* [5,27,28]. As such, these strains show efficacy for rescuing GIT inflammatory states. Although the current evidence as to the efficacy of probiotics to reduce uremic toxins is limited, the clinical evidence demonstrates that specific strains in a multiple-strain matrix configuration, in combination with prebiotics, may be most beneficial in reducing gut derived uremic toxins (Table 1). In addition, selecting probiotic species with known metabolic function, such as *Streptococcus thermophiles,* for metabolizing urea as a nitrogen growth source could contribute to reducing uremia. 

## 5. Commensal GIT Bacteria, Cellular Signaling and Macromolecular Oxidative Changes

Over the last decade oxidative stress has been proposed to play a major role in the development of co-morbid conditions such as CVD among renal failure patients [29,30], advocating that antioxidant strategies should become part of the treatment for pre-dialysis renal failure [31]. Additionally, it has been proposed that uremic toxins and oxidative stress play significant roles in the development of uremia and its complications [12]. Thus, the concept of oxidative stress being a major deleterious player in all manner of situations has been massively supported by a vast literature [31,32,33]. The data presented in these reports has formed the basis for pre-clinical and clinical trials of antioxidant therapies [34,35], most of which have disappointing outcomes. We assert that the conclusion of a need for antioxidant therapy is based on misinterpretation of these data. Reactive oxygen species (ROS) are central to normal metabolism. They give rise to physiological levels of peroxide, which then acts as a second messenger in maintaining normal physiological function [36]. Thus, ROS at physiological levels are essential for health.

**Table 1:** Clinical trials investigating the administration of probiotics and prebiotics in chronic kidney disease (adopted and modified from Vitetta and Gobe [2]).

Clinical Trial Type [Reference] Probiotic Strains or Prebiotics used Dose administered	Participant Type Location (*n* = Number) Study Duration	Clinical Trial Results
Open label pilot study [37] *L. acidophilus* Dose: not specified	Haemodialysis USA (*n* = 8) One course administered with the time-course undefined	Probiotic treatment was effective in: ↓serum dimethylamine from 224 ± 47 to 154 ± 47 μg/dL (*p* < 0.001) ↓nitrosodimethylamine 178 ± 67 ng/Kg (untreated) to 83 ± 49 ng/Kg (after treatment) (*p* < 0.05); Probiotic treatment: modified metabolic actions of small bowel bacterial overgrowth ↓*in vivo* generation of toxins and carcinogens
Prospective pilot DBRPC crossover trial [38] *S thermophilus* *L. acidophilus* *B longum* Dose: KIBOW biotics 90 × 10^9^ CFU/day	CKD stages 3 and 4 USA (*n* = 10) Canada (*n* = 113) Nigeria (*n* = 115) Argentina (*n* = 8) 6 months	Probiotic treatment: ↓ BUN (29 patients (63%, *p* < 0.05)); ↓Creatinine (20 patients (43%, *p* > 0.05)); ↓Uric acid (15 patients (33%, *p* > 0.01)) subjects expressed overall improvement in QoL (86%, *p* < 0.05);
Prospective pilot DBRPC crossover trial [39] S*. thermophilus* KB27 *L. acidophilus* KB31 *B. longum* KB35 Dose: KIBOW biotics 90 × 10^9^ CFUs/day	CKD stages 3 and 4 Canada (*n* = 13) 6 months	↓BUN [probiotic (-2.93 mmol/L *versus* placebo (4.52 mmol/L) *p* = 0.002; ↓mean uric acid during probiotic period (24.70 μmol/L) *versus* placebo period (50.62 μmol/L) *p* = 0.050; no significant change serum creatinine no gastrointestinal/infectious complications improved QoL
Single centre, non-randomized, open-label phase I/II study [40] Escalating dose regimen of oligofructose-enriched inulin.Dose: 10 g b.i.d.	Haemodialysis Belgium (*n* = 22)	20% ↓p-cresyl sulfate serum concentrations at 4 weeks (intention to treat, *p* = 0.01; per protocol, *p* = 0.03); ↓p-cresyl sulfate generation rates were reduced (*p* = 0.007); no significant changes in indoxyl sulfate generation rates/serum concentrations
Open label single arm study [41] *L casei* Shirota *B breve* Yakult + galacto-oligosaccharides (as prebiotics) Dose: 1 × 10^8^ CFU/mL probiotics and 1.67 g of prebiotic t.i.d. for 2 weeks.	Haemodialysis Japan (*n* = 8) 4 weeks preceded by 2 weeks of pretreatment observation	Pretreatment observation period: haemodialysis patients with a high serum p-cresol level tended to have hard stools with difficulty in defecation; SYN treatment: stool quantity increased significantly hard, muddy or soft stools tended to be replaced by normal ones significant ↓serum p-cresol

Notes: BUN: blood-urea-nitrogen; CFU: Colony-Forming Units; HD: Hemodialysis; QoL: Quality of Life. SYN: Synbiotic (probiotic + prebiotic); DBRPC: Double Blind Randomized Placebo Controlled; b.i.d.: twice per day; t.i.d.: three times per day.

The investigations on protein albumin thiol oxidations and serum protein carbonyl formations indicate that these biochemical events progressively increase with advancing stages of CKD [33], leading to the conclusion that there exists a close association between oxidative stress and carbonyl formation and that there is a correlation with carbonyl formation and renal dysfunction among pre-dialysis CKD patients [33]. However, there are no reported clinical trials that support this conclusion; indeed, studies on the protective antioxidant role of administered alpha lipoic acid for the prevention of contrast-induced nephropathy in diabetic patients demonstrated no benefit [34]. A recent systematic review and commentary reported that antioxidant therapy with vitamin C does not reduce the risk of death or cardiovascular events overall in CKD, but that there may possibly be benefits in people with more advanced kidney failure [35]. We [36] have previously considered and challenged the commonly held view that proteins are randomly oxidized in an uncontrolled process by superoxide anion, hydrogen peroxide, nitric oxide and peroxynitrite, thereby contributing directly to the development of chronic diseases and the aging process. We concluded that this concept is not tenable and these data misrepresent the stringently regulated cellular role of ROS in redox metabolism.

The oxidation of protein amino acid residues, since their discovery some decades ago, has been almost universally reported as leading to protein inactivation and requiring mandatory proteolysis to prevent their deleterious cellular accumulation. It is clear that oxidatively modified proteins do not simply arise as the result of random oxidative damage (hydroxylations of various amino acid residues, sulfoxidation of methionines, nitrosylations of sulfydryl groups, and so on). There is an increasing number of situations where free radical protein modifications can be shown to be part of normal cellular regulatory signaling activity. To support these conclusions, some examples follow.

### 5.1. Specific Protein Oxidations

(i) One of the most sensitive amino acids to oxidation is methionine, being converted to methionine sulfoxide. This phenomenon is commonly cited as an example of random oxidative damage to proteins. The following example would bring such an overriding conclusion into serious question. Calmodulin function and its regulation by superoxide anion/hydrogen peroxide oxidation of specific methionine residues is well documented [42]. The oxidation of only two of the seven specific methionine residues (144 and 145) of calmodulin is involved in the process of down-regulating plasma membrane Ca^++^ATPase. Using genetically engineered calmodulin in which the two methionines (144,145) were replaced by glutamines, it was shown that oxidation of the remaining methionines did not significantly down-regulate calmodulin-plasma membrane-Ca^++^ATPase activation [43]. It has also been reported from the same laboratory [44] that methionine sulfoxide reductase can act reductively to restore the ability of oxidized calmodulin to regulate plasma membrane-Ca^++^ATPase. These results showed that superoxide anion and/or hydrogen peroxide are functioning as part of the controlled regulation of the calmodulin-plasma membrane-Ca^++^ATPase complex. Furthermore, proteasomal degradation of oxidized calmodulin, when and where it occurs, is part of the normal process of regulated protein turnover. Protein turnover is rigidly controlled: while some proteins turn over in minutes, others take hours or longer, yet all these proteins are part of a system tightly regulated by the ubiquitin/proteasome system.
(ii) The turnover of the hypoxia-inducible factor-alpha (HIFα) and its proteasome degradation is clearly regulated by hydroxylation of its prolyl residues [45]. This is an ordered process involving signaling by the free radical system comprised of superoxide anion, nitric oxide and peroxynitrite.
(iii) Bota *et al.* [46,47] have reported that mitochondrial aconitase is preferentially oxidatively modified and inactivated, and that the ATP activation of the mitochondrial Lon protease specifically acts to degrade the oxidized inactivated enzyme. The authors interpret their results as demonstrating the toxicity of ROS. In direct contrast, we believe that that their results demonstrate how tightly regulated is the formation of ROS and its directed activity in regulating the metabolome. The controlled specific degradation of aconitase (among the hundreds of mitochondrial proteins) to regulate citric acid cycle activity is an excellent example of the regulatory role that ROS play in the modulation or control of the metabolome, and that ROS do not randomly contribute to the damage or degradation of cellular metabolic processes.
(iv) Consider the nitrosylation of sulfydryl groups, proposed as a damaging phenomenon. We have previously referred to and cited the hemoglobin system as a remarkably regulated machine, finely tuned allosterically for the carriage of the daily massive amounts of inhaled oxygen from lungs to cells [36]. It is now recognized in textbooks that hemoglobin undergo subtle but critical changes as result of sequential reactions with dioxygen, protons and CO_2_ to regulate the delivery of oxygen to the tissues. As part of this process, it has been recognized relatively recently that NO^−^ participates in the regulation [48]. In the cyclic oxygen carriage by hemoglobin, NO^−^ reacts with the b subunit ferrous ions. Subsequently, on the b subunit binding of dioxygen, the NO^−^ is displaced to nitrosylate the cys 93 thiol group of the hemoglobin b subunit. These changes are accompanied by an allosteric change from the T (tense) to the R (relaxed) form. The various allosteric changes which hemoglobin undergoes are now recognized as of the utmost importance to hemoglobin function. They are the outcome of over 80 years study (that includes the Bohr effect/Perutz X-ray structural studies/the detailed effector allosteric inducers, namely H^+^, CO_2_, O_2_, NO^−^, 2;3-bisphosphoglycerate/and the allosteric positive and negative co-operative changes that occur) [36]. What is important here is that the comparatively recently recognized NO^−^ nitrosylation of hemoglobin is part of the normal physiological transport of oxygen delivery to tissues; nitrosylation of proteins is not conditionally deleterious. Parenthetically it may be added that superoxide anion continually formed in small amounts during the process oxidizes hemoglobin to met hemoglobin in the order of a steady state amount of 1%–3%. The met hemoglobin formed is itself continually reduced back to hemoglobin by erythrocyte met hemoglobin reductase to maintain regulated oxygen homeostasis.
(v) Farout and Friguet [49] have considered that there is an age-related deleterious accumulation of oxidized proteins resulting from impaired redox homeostasis and proteolysis. Further, they consider that changes in proteasome structure with increasing age and dysfunction of the proteasome leads to an exacerbated accumulation of oxidatively modified proteins due to their impaired proteolysis. Somewhat contrary to this interpretation, it has been reported when cellular proteasome activity is inhibited, the resultant decrease in its activity leads to a concerted increase in cellular synthesis of the proteasome (the phenomenon of hormesis) [50]. Husom *et al.* [51] have reported an increase in the 20S proteasome in aged rat skeletal muscle, albeit with some change in function.

We suggest that the proteasome activity and changes in structure with increasing age and organ dysfunction (for example, in the kidneys and the gut epithelium) be viewed from a different perspective. The proteasome system makes a major demand on the available cellular ATP and will become increasingly dysfunctional in the absence of sufficient ATP substrate. Central to any consideration of aging and its outworking is the universally recognized decline in bioenergy capacity with age from increasingly dysfunctional mitochondria (DNA mutations and deletions). Arising from this consideration, declining ATP availability leads to declining proteasome function which contributes to the multisystem aging process, that includes single organ dysfunction (e.g., the kidneys), albeit not as a primary effector and not as a direct result of oxygen radical damage to proteins. Furthermore, with the increasing understanding of the upstream regulation of the superoxide anion/hydrogen peroxide second messenger system, it is becoming increasingly apparent that they play a major role in the ordered regulation of proteolysis and protein homeostasis; and that the damage process is far from random.

### 5.2. The Antioxidant Effect

Vitamin C has long been promoted as an outstanding antioxidant and of benefit in the prevention or amelioration of age-associated diseases proposed as arising from oxygen radical damage. There is no doubt that vitamin C is an essential nutritional supplement required for normal mammalian function but it has yet to be demonstrated by clinical trial that it has any role as a meaningful therapeutic antioxidant. 

Ascorbic acid plays an essential co-enzyme oxido-reductase role in the hydroxylations of pro-collagen (pro-collagen trimer formation and release from the endoplasmic reticulum), dopamine (to give rise to norepinephrine) and hypoxia inducible factor-alpha (HIFa) (regulation). Ascorbate occurs in high concentration in the adrenal and pituitary glands but it is not evenly distributed throughout mammalian tissues. Its occurrence is low in tissues such as skeletal muscle, testes, thyroid and lung [52], so that it would not constitute a general endogenous tissue antioxidant, if that were its proposed antioxidant role. Recently, it was reported that administered ascorbate acts as a pro-oxidant during surgical ischemia-reperfusion [53]. There is no convincing clinical evidence for ascorbic acid acting beneficially in mammals as an antioxidant. On the contrary, in large doses, it may act as a pro-drug for the production and delivery of H_2_O_2_ to tissues, especially as has been reported for the treatment of some cancers [54].

### 5.3. Commensal and Probiotic Bacteria Mechanism of Action

CKD has been recently linked to severe disruption of the gut epithelial tight junction barrier [55,56] with oxidative stress, the reported primary factor that drives metabolic abnormalities that contribute to CKD development [56]. It is extensively reported that probiotics can temper a range of GIT physiological functions, including control over immune responses, epithelial barrier function and cellular proliferation [18]. A recent study has demonstrated that some genera of human GIT bacteria can induce a rapid increase of ROS, eliciting a physiological response through the activation of epithelial NADPH oxidase-1 (Nox1) [57,58]. In addition, reports site *in vitro* experiments with epithelial cells that, when co-cultured with specific probiotic bacteria, show an increased and rapid oxidation reaction of soluble redox sinks, namely glutathione and thioredoxin [57,58] that indicate the presence of a regulated process. This effect was demonstrated as an increase in the oxido-reductase reaction of transcriptional factor activations such as nuclear factor kappa B (NFκB), NrF2 and the antioxidant response element, reflecting a cellular response to increased ROS production that is regulated [57,58]. This effect must be decisive in order to elicit a restrained anti-infective response with a minimal chance of pro-inflammatory damage to the tissue. These reactions define potent regulatory effects on host physiological functions that include immune function and intracellular signaling. 

The reported mechanisms of action of probiotics are similarly aligned, acting to enhance the epithelial barrier and increase bacterial adhesion to the intestinal mucosa, with an attendant inhibition of pathogen adhesion to the competitive exclusion of pathogenic microorganisms [57,58,59,60,61,62,63]. Furthermore, probiotic strains have also been reported to generate a range of anti-microbial substances and to positively affect and modulate immune system function. Lee [60] has reported that the enteric commensal bacteria by rapidly generating ROS negotiate an acceptance by the GIT epithelia. Different strains of commensal bacteria can elicit markedly different levels of ROS from contacted cells. *Lactobacilli* are especially potent inducers of ROS generation in cultured cells and *in vivo*, though all bacteria tested have some ability to alter the intracellular oxido-reductase environment [59]. Yan [61] has reported that there are soluble factors that are produced by strains of *lactobacilli* that are capable of mediating beneficial effects in *in vivo* inflammatory models. This result expands our understanding that there are ROS-stimulating bacteria that possess effective specific membrane components and or secreted factors that activate cellular ROS production to maintain homeostasis. 

It has been reported that redox signaling by microbial ROS formation is in response to microbial signals via formyl peptide receptors and the gut epithelial NADPH oxidase 1 (Nox1) [58]. As we have previously documented [36], ROS generated by Nox enzymes have been shown to function as essential second messengers in multiple signal transduction metabolic pathways through the rapid and transient oxidative inactivation of a distinct class of sensor proteins bearing oxidant-sensitive thiol groups. These redox-sensitive proteins include tyrosine phosphatases that attend as regulators of the MAP kinase pathways, focal adhesion kinase [36,58]. These reports focus our understanding on the importance of second messenger functionality for the maintenance of homeostasis and brings into serious question the annulment of ROS by antioxidant supplements for the amelioration of chronic diseases such as CKD. The established importance of recent investigations regarding probiotic/microbial-elicited ROS teaches that stimulated cellular proliferation and motility is strictly controlled and is a regulated signaling process for proper innate immunity and gut barrier functionality [59,62,63]. The observations that the vertebrate epithelia of the intestinal tract supports a tolerable low-level inflammatory response toward the GIT microflora can be viewed as an adaptive activity that maintains homeostasis [64].

## 6. Discussion

From the time of birth, humans begin to experience a bacterially directed and regulated control of GIT metabolic activities. The primary mediator involves an induced pro-inflammatory flux by a fleet of bacteria that assault all mucosal surfaces as well as the skin, thus initiating effects that eventually provide the infant with an immunological profile that is concordant with immune tissue maturation and gut barrier functionality. These effects occur beneath an emergent response sensor of immune system surveillance and antigenic tolerance capabilities. Over time, continuous and regulated interactions with environmental as well as commensal microbial and viral antigens lead to an adapted and maintained symbiotic state of tolerance, especially in the GIT—the organ site of the largest microbial biomass. However, the perplexing and much-debated surprise has been that all microbes need not be targeted for destruction. The advent of sophisticated genomic techniques has led to microbiome studies that have clarified the critical and important biochemical activities that commensal bacteria provide to ensure continued GIT hormesis.

While the induced production of ROS is a well-studied process in phagocytic cells arising through the stimulation of formyl peptide receptors and the subsequent activation of NADPH oxidase 2, ROS are also similarly elicited in other cell types that include the GIT epithelia. Historically, ROS signaling generated by the action of Nox enzymes [65,66] are reported to be induced responses to bacteria in all forms of multicellular life ranging from plants to amoebae to humans, representing a primal system of microbial attentiveness and regulated control of the metabolome rather than a process leading to random macromolecular damage. Amidst a huge literature that supports the overproduction of ROS and oxidative stress, recent reviews have described how intracellular and extracellular thiol-disulfide redox homeostasis is central to intestinal function and integrity [58,67], important factors that have been reviewed as causal in CKD [2]. Insights from fruit fly studies have shown that intestinal homeostasis is successfully achieved, albeit in part, by the integration of a complex set of mechanisms that controls pathogen activity while tolerating the indigenous microbiota [68]. Probiotic bacteria are microbial species that comprise the indigenous intestinal flora. 

The theme running throughout this critical review and commentary is aligned to end-stage CKD (type 5). The aim was to build a hypothesis that would introduce a biologically plausible therapeutic (with probiotics or without prebiotics) to treat early in CKD development, potentially stage 3a, and therefore prevent the progression to end-stage disease. This is of particularly relevant and important, given that a recent study has demonstrated that colonic microbial activity may contribute to uremic solute production [69].

Numerous studies have reported the benefits of live probiotic cultures in disease states [18]. It would seem biologically plausible that maintaining the intestinal epithelial redox environment with probiotic bacteria that tolerate the host can be viewed as an essential requisite for the activities of strategic physiological processes. GIT-derived physiological functions include nutrient digestion, cell proliferation and apoptosis, and regulated pro-inflammatory and anti-inflammatory immune responses. Probiotics demonstrate properties that can promote and rescue deviations in intestinal redox metabolism through the activity of ROS in a similar manner as somatic cells signal metabolic function. Taken together, a growing body of evidence suggests that the microbiota can positively contribute to intestinal homeostasis and systemic physiology. 

We have here advanced a hypothesis on how the intestine perceives non-pathogenic bacteria, and how the microbiota mechanistically influences gut physiological biology. This review has described a fundamental, highly conserved oxido-reductase-based response of host epithelial cells to bacteria that may comprise an important component of the host microbial metabolome interactions that govern health and disease. A recent review has supported the use of probiotics in at least those patients with end-stage kidney disease [70], a concept that is very much aligned to the various documented adverse health sequelae and metabolic profiles of end-stage kidney disease [71].

Live probiotic cultures administered as a multi-strain probiotic supplement may plausibly lead to reductions in GIT uremia and improve CKD adverse outcomes. A recent 12-month clinical study of exercise training and lifestyle intervention in patients with CKD supports this view. The results were linked to better cardiorespiratory fitness, body composition, and diastolic function [72], parameters that other reports align these beneficial effects to the GIT microbiome and healthy physiological function [73]. 

The study and understanding of health-promoting probiotic bacteria is in its infancy; it is complex and intellectually and experimentally challenging. 
